# Functional roles of sialylation in breast cancer progression through miR-26a/26b targeting ST8SIA4

**DOI:** 10.1038/cddis.2016.427

**Published:** 2016-12-29

**Authors:** Xiaolu Ma, Weijie Dong, Zhen Su, Lifen Zhao, Yuan Miao, Nana Li, Huimin Zhou, Li Jia

**Affiliations:** 1 College of Laboratory Medicine, Dalian Medical University, Dalian, China; 2 Department of Clinical Laboratory, The First Affiliated Hospital of Dalian Medical University, Dalian, China; 3 Department of Biochemistry, Dalian Medical University, Dalian, China; 4 Graduate School, Dalian Medical University, Dalian, China; 5 Department of Microbiology, Dalian Medical University, Dalian, China

## Abstract

Sialylation is one of the altered glycosylation patterns associated with cancer progression. In this study, we investigated the *N*-glycan profiles of breast cancer patients and cell lines to reveal sialylation associated with breast cancer progression, and provided new evidences of miRNA-mediated sialylation. MALDI-TOF MS analysis revealed that *N*-glycans found in breast cancer tissues and breast cancer cell MDA-MB-231 featured increased levels of sialylation compared with adjacent tissues and normal breast epithelial cell MCF-10A. The expressional profiles of 20 sialyltransferase genes were then analyzed and found significantly different comparing breast cancer samples with adjacent tissues, and two breast cancer cell lines MDA-MB-231 and MCF-7 with different metastatic potential and MCF-10A cells. Tumor tissues and highly metastatic breast cancer cell line MDA-MB-231 exhibited higher levels of *ST8SIA4*. Knocking down *ST8SIA4* in breast cancer cell lines significantly inhibited their malignant behaviors including cell proliferation and invasion in a sialyltransferase-dependent manner. By applying bioinformatic approaches for the prediction of miRNA targeting 3′-UTR of *ST8SIA4*, we identified *ST8SIA4* as one of the miR-26a/26b-targeted genes. Further data analysis revealed the inversely related expression of ST8SIA4 and miR-26a/26b in breast cancer cells, tumor tissues and corresponding adjacent tissues. The ability of miR-26a/26b to interact specifically with and regulate the 3′-UTR of ST8SIA4 was demonstrated via a luciferase reporter assay. The forced expression of miR-26a/26b was able to induce a decrease of ST8SIA4 level and also to affect breast cancer cells progression, while altered expression of ST8SIA4 in breast cancer cells modulated progression upon transfection with miR-26a/26b mimics or inhibiter. Taken together, these results indicate that changes in the glycosylation patterns and sialylation levels may be useful markers of the progression of breast cancer, as well as miR-26a/26b may be widely involved in the regulation of sialylation machinery by targeting ST8SIA4.

Breast cancer is one of the most frequent malignant disease and primary cause of death in women worldwide.^1^ Therefore, it is extremely crucial to not only treat but also prevent this disease from becoming malignant. Malignant transformations are often associated with a deregulation of glycosylation processes, and in particular that of terminal sialylation in breast cancer.^2^ Lin *et al.*^3^ showed that the cell surface *α*2, 6-sialylation contributed to cell–cell and cell–extracellular matrix adhesion of mammary carcinoma cells, and inhibition of sialytransferase ST6Gal-I level reduced the metastatic capacity of mammary carcinoma cells. Yuan *et al.*^4^ found that the desialylation of *α*2, 6-sialylated integrins increased adhesion of breast cancer cell MDA-MB-231 to ECM without altering integrin expression. Lee *et al.*^5^ demonstrated that highly sialylated and fucosylated complex-type *N*-glycans were elevated in six human epithelial breast cells relative to normal mammary epithelial cells. In consequence, sialylation and changes of sialyltransferases (STs) can be useful for breast cancer diagnosis and progression, but can as well be targets for therapeutic strategies.

Sialylation is governed by STs and sialidases. Sialic acids are transferred from a donor substrate to terminal positions of glycoprotein and glycolipid carbohydrate groups by STs.^6^ STs are categorized into four families on the basis of the carbohydrate side chain they synthesize, namely ST3Gal (*α*2, 3-ST), ST6Gal (*α*2, 6-ST), ST6GalNAc and ST8Sia (*α*2, 8-ST).^7^ On the other hand, their removal from glycan chains is catalyzed by sialidases. The activity of these enzymes is believed to affect the conformation of glycoproteins, and therefore contribute to either increased recognition or masking of biologically relevant sites in molecules and cells.^8^ High expression of ST3Gal-II was associated with poor clinical outcome in breast cancer patients treated with chemotherapy.^9^ Inhibition of ST6Gal-I expression reduced the metastatic capacity of breast cancer cells MDA-MB-435.^3^ ST6GalNAcI expression was sufficient to enhance the tumorigenicity of MDA-MB-231 cells.^10^ ST6GalNAcV was identified as one of the genes overexpressed in breast cancer cell populations selected for their ability to produce brain metastasis.^11^ ST8SiaI was also overexpressed in estrogen receptor-negative tumor samples of different subtype breast cancer.^12^ Although STs have an important role in breast cancer progression, investigating the regulation of sialylation by microRNAs (miRNAs) remain unknown.

MiRNAs are small noncoding RNAs implicated in the modulation of diverse biological processes through a mechanism based on the repression of protein translation or degradation of messenger RNAs.^13, 14, 15, 16^ Recent studies have identified several deregulated miRNAs (e.g. miR-147, miR-340 and miR-1296) in breast cancer tissues or cells, and revealed their potential roles in breast cancer progression by contributing to cell proliferation, survival and metastasis.^17, 18, 19^ However, the molecular basis for these inhibitory effects of miR-26a/26b targeting ST8SIA4 has not been fully elucidated yet in breast cancer.

On the basis of these earlier observations, our objective here was to evaluate the alteration of *N-*glycan profiles and sialylated *N*-glycans in adjacent and tumor tissues from breast cancer patients and breast cancer cell lines. Expressional levels of ST genes were measured between the tumor samples and adjacent tissues, and breast cancer cell lines. In addition, ST8SIA4 was also one of the miR-26a/26b-targeted genes. We described here a novel mechanism for the changes of breast cancer-associated *N*-glycan and sialylation and the suppression of ST8SIA4 by miR-26a/26b in breast cancer progression.

## Results

### MALDI-TOF MS analysis for *N*-glycan profiling of breast cancer tissues and cell lines

**Figure 1:**
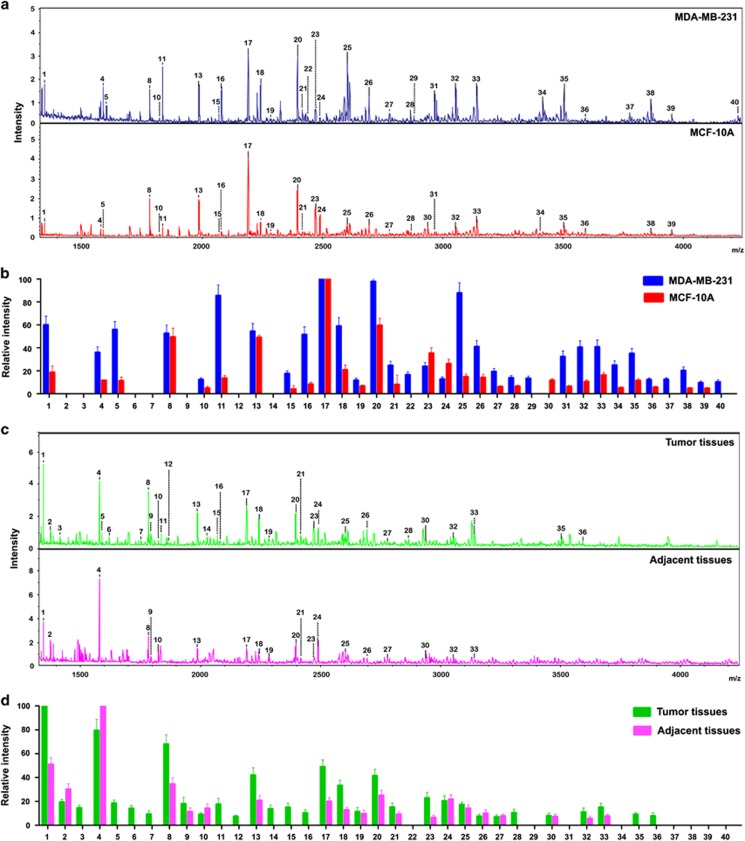
Differential *N*-glycan composition of breast cancer tissues and cell lines. (**a**) MALDI-TOF MS spectra of permethylated *N*-glycans released from MDA-MB-231 and MCF-10A cells, respectively. (**b**) Histograms of relative intensities of the differential *N*-glycan signals from the both cell lines were observed. The histograms represented only signal intensities but not the quantities. (**c**) MALDI-TOF MS spectra of *N*-glycans from breast cancer and adjacent tissues were shown, respectively. *N*-glycans were released by PNGase F and permethylated. (**d**) Histograms of relative intensities of the differential *N*-glycan signals from the both cases were observed. Values are mean±S.D. for three permethylated samples from *N*-glycan samples. The signals indicated with Arabic numerals are summarized in Table 1

Whole *N*-glycans of highly metastatic breast cancer cell MDA-MB-231 and immortalized breast epithelial cell MCF-10A were analyzed and identified by MALDI-TOF MS spectrometry (Figure 1a). As summarized in Table 1, 33 kinds of glycoforms were detected and quantified reproducibly in both cell lines. Histograms of *N*-glycan compositions produced from three MALDI-TOF MS replicates were shown in Figure 1b. When the full portraits of *N*-glycan diversity of both cell lines were represented quantitatively, it seems likely that high-mannose-type *N*-glycans (peaks 4, 8, 13, 17 and 20; Table 1) were major components. However, it was also clearly suggested that expression levels of four *N*-glycans (peaks 22, 29, 37 and 40) were identified exclusively in MDA-MB-231 cells, and all of which were sialylated glycoforms (Table 1). Furthermore, MDA-MB-231 cells also exhibited upregulated high-level expression of glycoforms (peaks 1, 4, 5, 10, 11, 15, 16, 18, 21, 25, 26, 27, 31, 32, 33, 34, 35, 38 and 39, >2-folds) compared with MCF-10A cells, and eight sialylated *N*-glycan compositions (peaks 25, 27, 32, 33, 34, 35, 38 and 39). Peak 24 (not containing sialylated *N*-glycan) clearly showed a significant increase in MCF-10A cells (>2-folds).

We then applied the above method to analyze *N*-glycans from breast cancer tissues and corresponding adjacent tissues. Figure 1c showed MALDI-TOF MS spectra of whole *N*-glycans enriched by glycoblotting at both cases. A total of 32 *N*-glycans were identified and the *N*-glycan composition was summarized in Table 1. High-mannose glycans (peaks 4, 8, 13, 17 and 20) were observed in both tissues (Table 1). The peaks at 3, 5, 6, 7, 11, 12, 14, 15, 16, 28, 35 and 36 were exclusively detected only in the tumor group, and peak 35 was shown to be sialylated *N*-glycan (Figure 1d and Table 1). Furthermore, the expression level (peaks 13, 17, 18, 23, 32, and 33) was increased in the tumor group (>2-fold) compared with the adjacent tissue group, and peaks 23 and 32 were shown to be sialylated *N*-glycans (Figure 1d and Table 1). Notably, the peaks corresponding to sialylated glycoforms were often observed in tumor tissues and breast cancer cells.

### Identification of ST genes differentially expressed in breast cancer tissues and cell lines

**Figure 2:**
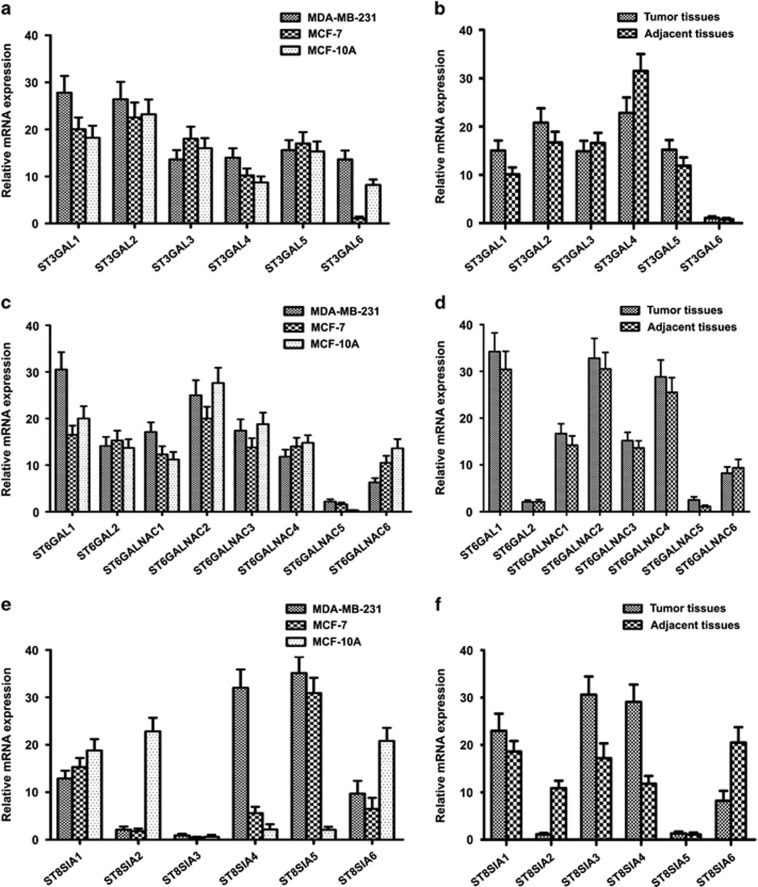
Differential expression of ST genes in breast cancer tissues and cell lines. The mRNA levels of ST genes analyzed by qRT-PCR. The relative amount of gene mRNA level was normalized to glyceraldehyde 3-phosphate dehydrogenase (GAPDH) level. (**a**, **c** and **e**) Relative intensities ratio of the ST gene signals from MDA-MB-231, MCF-7 and MCF-10A cell lines were observed. (**b**, **d** and **f**) Relative intensities ratio of the ST gene signals from breast cancer and adjacent tissues were observed. Data are the mean±S.D. of triplicate determinants

The purpose of these experiments was to identify altered expression patterns of ST genes that regulate sialylated glycoforms, by agnostically analyzing 20 ST gene expression levels in a panel of breast cancer cell models. In comparison with MCF-10A cells, the aggressive breast cancer cell line MDA-MB-231 showed an increased number of differentially expressed genes including *ST6GALNAC5* (7.33-folds), *ST8SIA4* (14.81-folds) and *ST8SIA5* (3.11-folds) (Figures 2c and e). Only slight difference was observed in the level of *ST3GAL1* (1.53-folds), *ST3GAL4* (1.61-folds), *ST3GAL6* (1.66-folds), *ST6GAL1* (1.56-folds), *ST6GALNAC1* (1.53-folds) mRNA in the both cell lines (Figures 2a and b). *ST6GALNAC6* (2.16-folds), *ST8SIA2* (10.86-folds) and *ST8SIA6* (2.14-folds) were also significantly upregulated in the MCF-10A cells (Figures 2c and e). In addition, the data revealed that expressional levels of *ST3GAL6* (12.34-folds) and *ST8SIA4* (5.71-folds) were upregulated in the highly metastatic breast cancer cell line MDA-MB-231 compared with the slightly metastatic breast cancer cell line MCF-7 (Figures 2a and e).

Subsequently, these 20 ST genes were measured in breast cancer and adjacent tissues. Of these, three genes have higher expression in the tumor tissues including *ST6GALNAC5* (2.31-folds), *ST8SIA3* (1.78-folds) and *ST8SIA4* (2.47-folds) (Figures 2d and f), suggesting that breast cancer expressed high levels of *α*2,6- and *α*2,8-linked sialylation. In addition, the data revealed that expressional levels of *ST8SIA2* (9.63-folds) and *ST8SIA6* (2.50-folds) were upregulated in the adjacent tissues (Figure 2f). Furthermore, the expression of ST mRNA was also normalized using *β*-actin (a second endogenous PCR normalizer) as control. These results were shown in Supplementary Figure 1.

As a potential candidate target, *ST8SIA4* was highly expressed in the aggressive MDA-MB-231 cells, but was slightly detectable in the MCF-7 and MCF-10A cells (Figure 2e). Interestingly, we also found that *ST8SIA4* was one of the most notable genes that show higher expression patterns in breast cancer tissues. In the current study, ST8SIA4 was chosen for further investigation. Collectively, these findings indicate a clear correlation between derived *N*-glycan traits based on MS data and corresponding expression patterns of STs that involved in the synthesis of sialylated glycans.

### Expression of ST8SIA4 is associated with breast cancer metastasis

**Figure 3:**
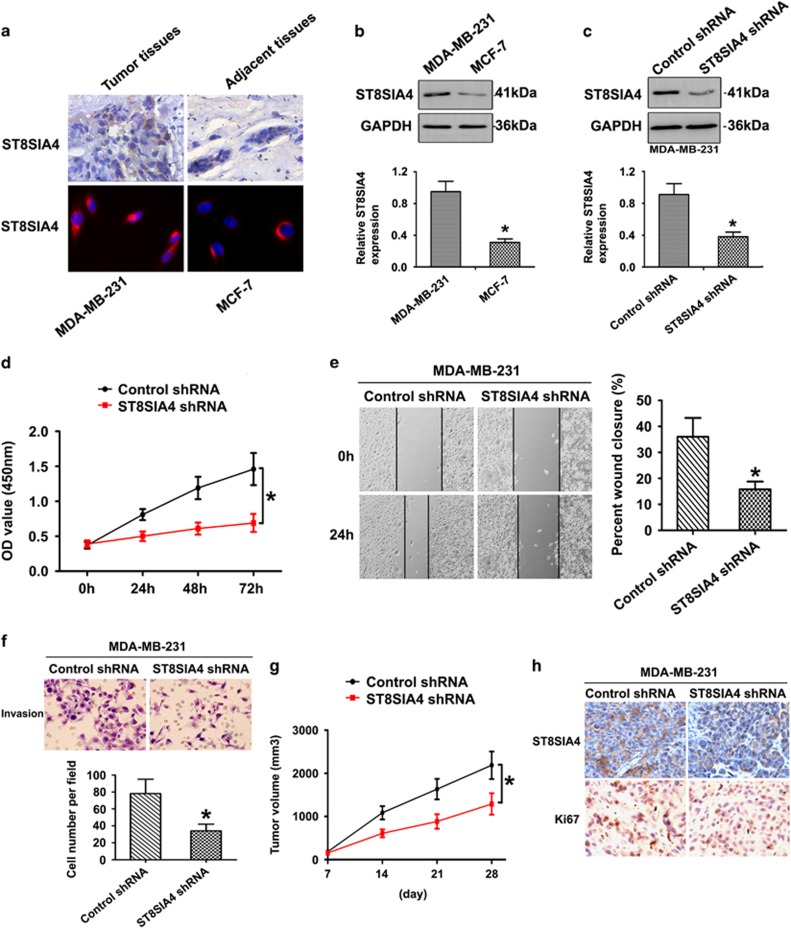
Silencing of ST8SIA4 inhibits the progression of MDA-MB-231 cells both *in vitro* and *in vivo*. (**a**) The ST8SIA4 expression from breast cancer tissues and MDA-MB-231 cells was obviously compared with that in adjacent tissues and MCF-7 cells by IHC and immunofluorescence staining. Red fluorescence: ST8SIA4; DAPI staining for nuclear DNA. (**b**) ST8SIA4 protein levels were increased notably in MDA-MB-231 cells compared with MCF-7 cells by western blot analysis. (**c**) ST8SIA4 transcript was decreased apparently in MDA-MB-231 cells by short hairpin RNA (shRNA) treatment. The distinct reduction of ST8SIA4 was observed at the protein level by western blot analysis. (**d**) Growth curves of MDA-MB-231 ST8SIA4 shRNA cells were compared with control cells with the CCK-8 assay (*P<0.05). (**e**) The ability of migration and invasion was compared in MDA-MB-231 ST8SIA4 shRNA and control shRNA cells based on wound healing (*P<0.05). (**f**) *In vitro* ECMatrix gel analysis was performed to compare cell invasion between MDA-MB-231 ST8SIA4 shRNA cells and control group (*P<0.05). (**g**) A decrease of mean tumor volume in mice group with MDA-MB-231 ST8SIA4 shRNA tumors was observed, as compared with the control group (*P<0.05). (**h**) Reduced regulation of ST8SIA4 and Ki67 was also shown by IHC staining in xenograft tumors derived from MDA-MB-231 ST8SIA4 shRNA cells (× 400). Values shown are mean±s.d. from three independent experiments

To determine the clinical significance of ST8SIA4, we examined its expression pattern in tumor samples collected from breast cancer patients. Immunohistochemistry (IHC) staining showed that ST8SIA4 expression was significantly upregulated in breast tumor tissues as compared with adjacent tissues (Figure 3a). Similarly, ST8SIA4 expression was also higher in MDA-MB-231 compared with that in MCF-7 cells (Figure 3a). As seen in Figure 3b, ST8SIA4 was highly expressed in MDA-MB231 cell line with high ability of metastasis.

We then examined whether the decrease in ST8SIA4 level is necessary for inhibition of breast cancer progression, and MDA-MB-231 cells were subjected to ST8SIA4 knockdown. As shown in Figure 3c, the expression of ST8SIA4 was significantly decreased in the protein level in shRNA-transfected cells compared with those in the controls (see Supplementary Figure 2a, ST8SIA4 shRNA3 cells showed lower expressional level of ST8SIA4, and ST8SIA4 shRNA3 was chosen for next experiments). As expected, Cell Counting Kit-8 (CCK-8) assays revealed that knockdown of ST8SIA4 repressed MDA-MB-231 cell proliferation (Figure 3d). In Figure 3e, MDA-MB-231 ST8SIA4 shRNA cells were subjected to a wound-healing assay. We observed longer distance in wound healing of ST8SIA4 shRNA cells compared with control groups. As advanced tumor stage often present with metastases, the invasion capacity of MDA-MB-231 cells transfected with ST8SIA4 shRNA *in vitro* was compared with that of parental control cells. As shown in Figure 3f, ST8SIA4 shRNA cells exhibited a significantly lower invasion potential compared with the control cells, suggesting a role of ST8SIA4 in metastasis.

The effects of ST8SIA4 on the tumorigenic potential of MDA-MB-231 cells were investigated *in vivo*. Results are shown in Figure 3g. Compared with mice injected with MDA-MB-231 cells transfected with control shRNA, mice injected with ST8SIA4 shRNA cells exhibited smaller tumors during the same time period, and the mean tumor volumes were significantly lower than the control group (*P<0.05). IHC staining also showed the reduced protein levels of ST8SIA4 and Ki67 in the tumor tissues (Figure 3h). Overall, our results demonstrated that ST8SIA4 indeed mediated tumor cell metastasis by modifying the sialylation profile in breast cancer cells.

### Direct regulation of ST8SIA4 by miR-26a and miR-26b

**Figure 4:**
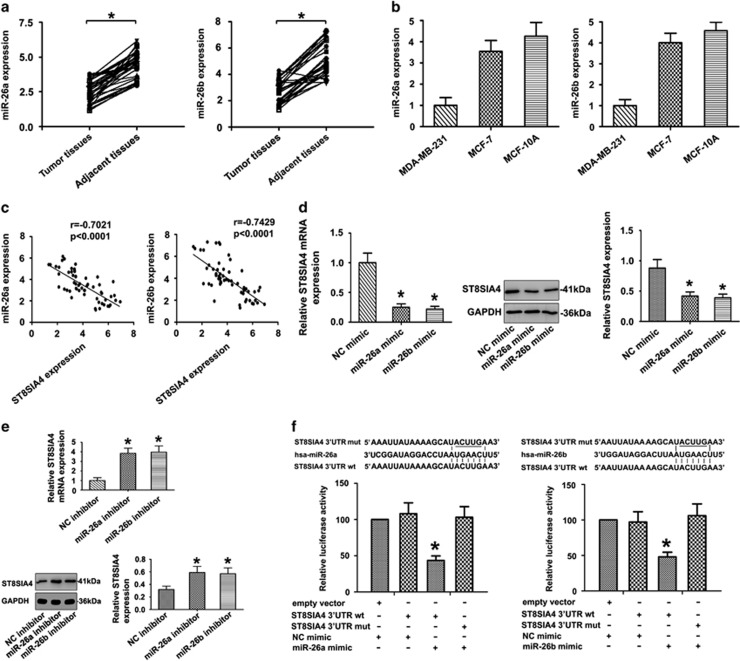
MiR-26a and miR-26b as negative regulators of ST8SIA4. (**a**) Paired comparisons between 29 breast cancer tissues and adjacent tissues were shown. miR-26a and miR-26b expressions were significantly decreased in cancer tissues compared with the corresponding adjacent tissues using qRT-PCR analysis. *P<0.05 from paired *t*-tests were provided. (**b**) The expression of miR-26a and miR-26b was examined by qRT-PCR in the MDA-MB-231, MCF-7 and MCF-10A cell lines. (**c**) Relationship between miR-26a and miR-26b levels and ST8SIA4 mRNA expression in 29 breast cancer tissues and 29 adjacent tissues. (**d**) ST8SIA4 was analyzed by qRT-PCR and western blot in MDA-MB-231 cells treated with miR-26a and miR-26b mimic (*P<0.05). (**e**) ST8SIA4 was analyzed by qRT-PCR and western blot in MCF-7 cells treated with miR-26a and miR-26b inhibitor (*P<0.05). (**f**) The nucleotide sequence of the target site of miRNAs in ST8SIA4 3′-UTR was shown; luciferase assay for the direct targeting of 3′-UTR of ST8SIA4 by miR-26a and miR-26b. The wild-type and mutant miRNA target sequences of T8SIA4 were fused with luciferase reporter and transfected into HEK 293T cells, transfected with miRNA mimic and NC mimic. The mean of the results from the cells transfected with the NC mimic was set at 100. Each bar represents the relative luciferase activity (*P<0.05). Values shown are mean±s.d. from three independent experiments. The expression of miR-26a/26b was normalized using U6 snRNA

It was known that miR-26a and miR-26b were significantly decreased in breast cancer tissues compared with normal tissues, implicating their potential roles in proliferation and motility of MDA-MB-231 and MCF-7 breast cancer cell lines.^20, 21^ To better explore and confirm the findings, we examined the expressional levels of miR-26a/26b in breast cancer samples and cell lines by quantitative real-time PCR analysis (qRT-PCR). MiR-26a/26b expression was significantly decreased in 29 breast cancer tissues compared with the 29 adjacent tissues (Figure 4a). Similar significant findings were identified by comparison of MDA-MB-231 cells with those of MCF-7 and MCF-10A cells (Figure 4b). In addition, the expression of miR-26a/26b was normalized using RNU48 (a second endogenous PCR normalizer) as control. These results were shown in Supplementary Figure 3. We then examined the relationship between miR-26a/26b and ST8SIA4 expression in 29 breast tissues and 29 adjacent tissues. As shown in Figure 4c, the ST8SIA4 mRNA expression appeared to be inversely correlated with the levels of miR-26a/26b (*P*<0.05), which was consistent with breast cancer cell lines.

We further investigated whether ST8SIA4 expression is regulated by endogenous miR-26a and miR-26b in breast cancer cell lines. MDA-MB-231 cells were transfected with miR-26a or miR-26b mimics, MCF-7 cells were transfected with the inhibitor and ST8SIA4 expression levels were measured. Overexpression of miR-26a and miR-26b remarkably reduced ST8SIA4 expression at both the mRNA and protein levels in MDA-MB231 cells (Figure 4d). Conversely, inhibition of miR-26a and miR-26b significantly increased ST8SIA4 levels in MCF-7 cells (Figure 4e).

Next, we also looked for miR-26a/26b potentially targeting ST8SIA4 using miRNA target prediction databases to identify highly conserved putative miRNA binding sites in ST8SIA4 3′-UTR. To verify that miR-26a and miR-26b directly target the 3′-UTR of ST8SIA4, we constructed luciferase reporter gene linked to the ST8SIA4 3′-UTR (WT 3′-UTR), and carried out dual-luciferase assays. HEK 293T cells were co-transfected with ST8SIA4 3′-UTR luciferase reporter construct and miR-26a/26b or NC mimic. As expected, miR-26a/26b inhibited the luciferase expression of the ST8SIA4 WT 3′-UTR construct (*P*<0.05), which was de-repressed by mutating the miR-26a/26b seed sequence within the ST8SIA4 3′-UTR (Figure 4f). Our results confirm that miR-26a and miR-26b negatively regulate ST8SIA4 via directly targeting ST8SIA4 3′-UTR.

### MiR-26a/26b affects metastasis of MDA-MB-231 and MCF-7 cells by targeting ST8SIA4

**Figure 5:**
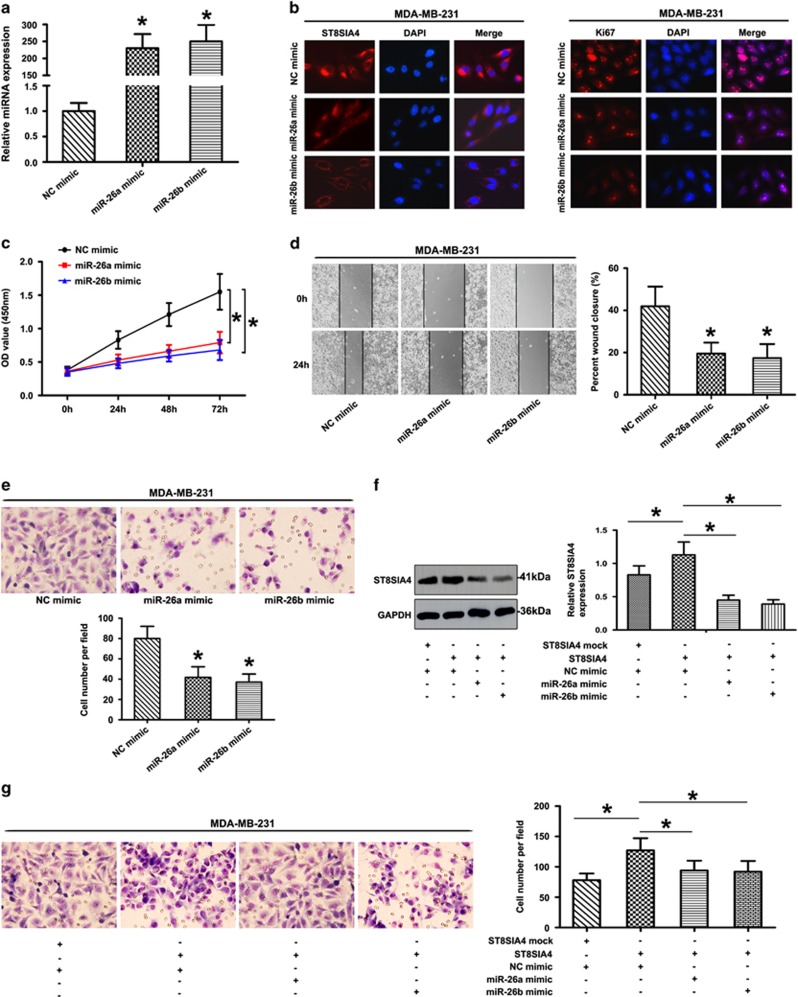
Effect of miR-26a and miR-26b mimics on cell progression in MDA-MB-231 cells. (**a**) The expression of miR-26a and miR-26b was studied by qRT-PCR in MDA-MB-231 cells transfected with the mimics (*P<0.05). (**b**) ST8SIA4 and Ki67 expression were detected by immunofluorescence staining in MDA-MB-231 cells treated with miR-26a or miR-26b mimic. Red fluorescence: ST8SIA4, Ki67; DAPI staining for nuclear DNA. (**c**) Transfection of miR-26a and miR-26b mimic in MDA-MB-231 cells inhibited cellular viability as revealed by CCK-8 assay (*P<0.05). (**d** and **e**) The ability of migration and invasion was compared in MDA-MB-231 cells with miR-26a mimic, miR-26b mimic or NC mimic based on wound healing (**d**) and transwell assays (**e**). The data were mean±S.D. of three separate transfections (*P<0.05). (**f** and **g**) MDA-MB-231 cells were co-transfected with target mRNA (ST8SIA4 mock or ST8SIA4) and miRNA mimics (NC mimic, miR-26a mimic or miR-26b mimic). Upregulation of ST8SIA4 inhibited the effects induced by miR-26a or miR-26b overexpression in MDA-MB-231 cells. Representative results of western blot (**f**) and transwell invasion assay (**g**) in MDA-MB-231 cells were shown (*P<0.05). Values shown are mean±S.D. from three independent experiments

To determine the impact of the miRNA-mediated downregulation of ST8SIA4 on breast tumor development, we assessed cell proliferation and invasion in breast cancer cell lines transfected with either control or miR-26a/26b mimics. As shown in Figure 5a, miR-26a/26b mimics were transfected into MDA-MB-231 cells, which exhibited the higher levels of miR-26a/26b expression. Immunofluorescence analysis showed that ST8SIA4 antigen expression was significantly decreased in MDA-MB-231 cells treated with miR-26a/26b mimics (Figure 5b). We then assessed proliferation also by detection of the expression of Ki67 antigen, which is a widely used marker for measuring the growth fraction of a given cell population. Ki67 expression was also decreased in the miR-26a/26b mimic groups (Figure 5b). The overexpression of miR-26a/26b significantly reduced MD-MB-231 cell proliferation efficiency compared with the negative control (Figure 5c). The wound-healing assay showed that the gap between the scorings was larger in mimic-treated group compared with that in control group in 48 h (Figure 5d). In cell invasion assays, exogenous upregulation of miR-26a/26b significantly inhibited the number of invasive cells significantly (Figure 5e). On the other hand, we found that overexpression of ST8SIA4 in MDA-MB-231 cells led to a marked decrease in the invasive cells and ST8SIA4 expression upon transfection with the miR-26a/26b mimics (Figures 5f and g).

**Figure 6:**
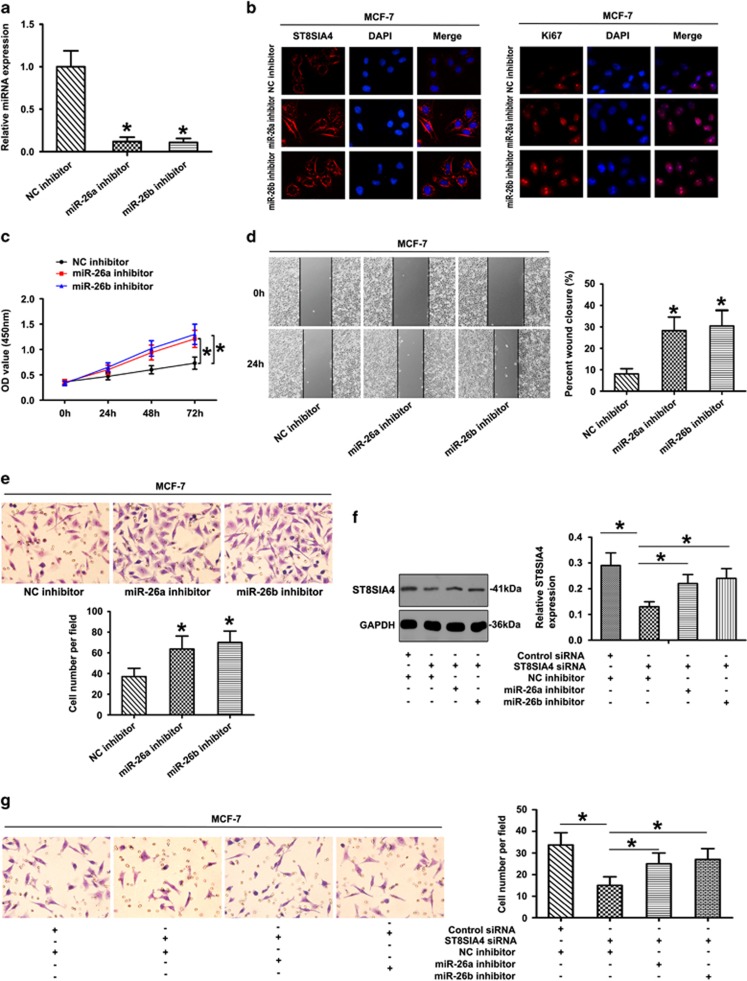
Effect of miR-26a and miR-26b inhibitors on cell progression in MCF-7 cells. (**a**) The expression of miR-26a and miR-26b was studied by qRT-PCR in MCF-7 cells transfected with the inhibitor (*P<0.05). (**b**) ST8SIA4 and Ki67 expression were detected by immunofluorescence staining in MDA-MB-231 cells treated with miR-26a or miR-26b inhibitor. Red fluorescence: ST8SIA4, Ki67; DAPI staining for nuclear DNA. (**c**) Growth curves of miRNA inhibitor-transfected cells were compared with NC inhibitor cells with the CCK-8 assay (*P<0.05). (**d** and **e**) Knockdown of miR-26a and miR-26b significantly increased the migration and invasion of MCF-7 cells by wound-healing (**d**) and transwell assays (**e**). (**f** and **g**) MCF-7 cells were co-transfected with target mRNA (control siRNA or ST8SIA4 siRNA) and anti-miRNA (NC inhibitor, miR-26a inhibitor or miR-26b inhibitor). The influence of miR-26a or miR-26b inhibitor was reversed by downregulation of ST8SIA4 expression in MCF-7 cells. Representative results of western blot (**f**) and transwell invasion assay (**g**) in MCF-7 cells were shown. The data were mean±S.D. of three separate transfections (*P<0.05)

Anti-miRNAs were transfected into MCF-7 cells followed by qRT-PCR analysis. As shown in Figure 6a, inhibitor of miR-26a/26b decreased the expression of miR-26a and miR-26b in MCF-7 cells. The ST8SIA4 and Ki67 expression were also increased in the miRNA inhibitor groups (Figure 6b). CCK-8 assays revealed that MCF-7 cells in the miRNA inhibitor groups had higher proliferative ability than controls (Figure 6c). Additionally, we observed shorter distance in wound healing of miRNA inhibitor groups compared with control groups (Figure 6d). We next asked whether inhibition of endogenous miR-26a/26b in MCF-7 cells would affect their motility. Indeed, expression of the miR-26a/26b antisense oligonucleotide increased the invasion of MCF-7 cells significantly (Figure 6e). Furthermore, the results showed that depletion of ST8SIA4 in MCF-7 cells by an RNAi-mediated silencing approach (see Supplementary Figure 2b, ST8SIA4 siRNA2 was chosen for next experiments) led to a marked increase in the invasive cells and ST8SIA4 levels upon transfection with the miR-26a/26b inhibitor (Figures 6f and g). In this regard, it would be conceivable that altered expression of miR-26a/26b targeting ST8SIA4 could downregulate ST8SIA4 levels to promote breast cancer progression, whereas ST8SIA4-mutant genetic background favored the miR-26a/26b-mediated cancer progression effect.

## Discussion

As MALDI-TOP MS has been used to effectively profile glycans in cancer,^22^ we expect that this method should be adaptable for *N*-glycan analysis in breast cancer tissues and cells. In this study, we revealed marked differences of *N*-glycans in breast cancer tissues, adjacent tissues and MDA-MB-231 and MCF-10A cells by MS analysis. This result corresponded with the current findings of *N*-linked glycan variations during breast cancer progression in the mouse model, as well as *N*-glycans from total human serum glycoproteins of breast cancer patients and controls were also profiled by MALDI-TOF-MS analysis.^23^ Furthermore, increasing changes of sialylated glycan intensities in MDA-MB231 cells (>2-folds in peaks 22, 25, 27, 29, 32, 33, 34, 35, 37, 38, 39 and 40) and breast cancer tissues (>2-folds in peaks 23, 32 and 35) were consistent with other reports in literature where sialylated glycans have been implicated in cancer invasion and metastases.^24, 25^ Thus, we believe this capability of profiling *N*-glycans and sialylated glycoforms spatially on breast cancer cell line and tissues will have significant application to tumor progression.

It is well known that changes in glycosylation of glycoproteins cause a change in their function and such phenomenon is correlated with cancerous transformation.^26, 27^ Aberrant glycosylation is one of the major hallmarks of cancer with altered gene expression signatures of STs. Mounting evidence demonstrated that the alterations of sialylation and the levels of ST activities derived from cancer cells were relevant to breast cancer invasion and metastasis.^28, 29^ Here, we indicated that the ST profiling patterns were significantly different in breast cancer tissues to adjacent tissues. Among these, the expression of *ST6GALNAC5*, *ST8SIA3* and *ST8SIA4* was significantly upregulated in breast cancer tissues compared with adjacent tissues, and ST8SIA2 and *ST8SIA6* were upregulated in the adjacent tissues. In addition, the expression profiles of ST gene family were shown to be remodeled in breast cancer cell lines.

MDA-MB-231 cells were characterized of higher levels of *ST6GALNAC5*, *ST8SIA4* and *ST8SIA5*, whereas MCF-10A cells expressed more *ST6GALNAC6, ST8SIA2* and *ST8SIA6*. Furthermore, the expressional levels of *ST3GAL6* and *ST8SIA4* were upregulated in MDA-MB-231 cells compared with MCF-7 cells. Studies of Cui *et al.*^29^ have shown that MDA-MB-231 had a higher expression of *α*2, 3-sialic acid residues compared with T-47D and MCF-7 depending on the mRNA levels of *α*2, 3-ST genes. In particular, we showed here that ST8SIA4 was specifically upregulated in breast cancer tissues and MDA-MB-231 cells, and thus we speculated that ST8SIA4 was a key factor in promoting malignant progression of breast cancer. Our findings here that silencing of *ST8SIA4* significantly inhibited MDA-MB-231 cell proliferation and invasion both *in vitro* and *in vivo* indicated an important role of ST8SIA4 in the stimulation of breast cancer cell progression. In line with our observations, much of the literature showed that ST8SIA4 was implicated as a potential glycan cancer marker.^30^ Additionally, ST8SIA4 has also been shown to confer multidrug resistance in human chronic myeloid leukemia.^31^ These studies imply that upregulation of ST8SIA4 was breast cancer progression-related. Clearly, further investigation was needed to elucidate the mechanistic roles of ST8SIA4 in breast cancer malignancy.

A few miRNAs that target glycosylation enzymes have been identified so far. MiR-424 targeted *N*-acetylglucosaminyltransferase that predicted a role for *β*-1,4 branched glycosylation in cell cycle progression in multiple mammary epithelial cell lines.^32^ MiR-199a targeted ST ST6GAL1 that added an 2, 6-linked sialic acid to the termini of *N*-linked glycans, and reduced its expression and the sialylation of Necl-2.^33^ MiR-200a targeted mRNA levels of ST ST3GAL3 and ST3GAL4, which potentially involved in antithrombin sialylation.^34^ Our recent results also showed that miR-224-3p directly targeted 3′-UTR of fucosyltransferase FUT4 mRNA and sensitized drug-resistant breast cancer cells to chemotherapeutics and reduced the growth rate of breast cancer xenografts *in vivo*.^35^

In our study, the levels of miR-26a/26b were measured in human breast cancer tissues and were significantly lower than in adjacent tissues. Accordingly, we also discovered that the expression of miR-26a/26b was reduced in MDA-MB-231 cells compared with those in MCF-7 and MCF-10A cells. Further experiments demonstrated that the regulated levels of miR-26a/26b were correlated with breast cancer cell progression both *in vitro* and *in vivo*. Similar observations have been described. MiR-26a/26b expression was significantly decreased in breast cancer tissues compared with normal tissues, and it was found associated with proliferation and motility in MDA-MB-231 and MCF-7 cell lines, indicating the diagnostic potential of miR-26a/26b in breast cancer.^20, 21^ Moreover, we detected that decreased levels of miR-26a/26b correlated with the upregulation of ST8SIA4 expression in the patients, as well as in cell lines. Moreover, silencing of the *ST8SIA4* gene in MCF-7 cells resulted in malignant behavior changes after miR-26a/26b inhibitor introduction. As far as we know, some studies have investigated the role of miR-26a/26b in breast cancer cells. It was reported that miR-26a negatively regulated the expression of PTEN in MDA-MB-231 and MCF-7 cells, which is involved in the regulation of cell migration of breast cancer cell lines.^36^ Overexpression of miR-26b inhibited MDA-MB-231 cell growth by targeting prostaglandin-endoperoxide synthase-2, suggesting its use as a potential therapeutic target for breast cancer.^37^ Our results additionally demonstrated that the function of miR-26a/26b in regulating breast cancer cell progression might be partially mediated by targeting ST8SIA4, because ST8SIA4 was highly correlated with cancer malignancy by regulating various biological processes.^31^

In conclusion, by analyzing the sialylated *N*-glycans and expressional patterns of ST genes in breast cancer patients and in cell lines, ST8SIA4 regulation elucidated the unusual properties of breast cancer biological behavior. In addition, we showed that miR-26a/26b was downregulated in breast cancer patients and invasive cells, and correlated with ST8SIA4 expression. Moreover, miR-26a/26b reconstitution contributed to improving the capability of breast cancer cell progression by regulating ST8SIA4. Prospective studies on larger cohorts of patients are required to substantiate their diagnostic role.

## Materials and methods

### Patients and tissue samples

The study was approved the Ethics Committee of the First Affiliated Hospital of Dalian Medical University. Written informed consent was provided by patients before any screening procedure and was required for the studies. All specimens were handled anonymously according to the ethical and legal regulations. In this study, 29 pairs of fresh breast cancer and adjacent tissues (>2 cm from tumor tissues) were obtained from the First Affiliated Hospital of Dalian Medical University (Dalian, China). The patients consisted of 29 women, with age ranging from 31 to 74 years (mean age of 48.6 years). None of the patients received any chemotherapy or radiotherapy ahead of surgery. All samples were confirmed with pathological diagnosis and were snap-frozen and stored in liquid nitrogen after collection. The clinical characteristics of breast cancer were as shown Supplementary Table 1.

### Cell culture

The breast cancer cell lines MDA-MB-231, MCF-7 and human epithelial breast cell line MCF-10A were purchased from the KeyGEN Company (Nanjing, China). MDA-MB-231 and MCF-7 cells was maintained in 90% Dulbecco modified Eagle's medium (DMEM; Gibco) supplemented with antibiotics (1 × penicillin/streptomycin 100 U/ml; Gibco) and 10% heat-inactivated fetal bovine serum (FBS; Gibco). MCF-10A, on the other hand, was maintained in DMEM-F12 media supplemented with hydrocortisone (0.5 *μ*g/ml), insulin (10 *μ*g/ml), hEGF (20 ng/ml) and 10% (v/v) FBS. All the cells were kept in a 37 °C incubator with 5% CO_2_.

### Membrane protein extract and preparation of *N*-glycans

For MALDI-TOF-MS analysis, membrane proteins were extracted from the tumor sample, adjacent tissue and MDA-MB-231 and MCF-10A cells using a CelLytic MEM Protein Extraction Kit (Sigma, St. Louis, MO, USA). The membrane protein concentration was measured with a Micro BCA Protein Assay Kit (Pierce, Rockford, IL, USA). For the release of *N*-glycans, three 100 *μ*g aliquots of lyophilized cell membrane proteins were first digested with trypsin (10 *μ*g) and chymotrypsin (10 *μ*g) dissolved in 25 mM ammonium bicarbonate (25 *μ*l) at 37 °C for 18 h. The digest was left in a water bath (85 °C for 5 min) and after cooling *N*-glycans were released from peptides by treatment with PNGase F enzyme (2 *μ*l; 6U) at 37 °C for 18 h followed by Pronase digestion (10 *μ*g) at 37 °C for 8 h. During the incubation time, the reaction sample was mixed occasionally. The released *N*-glycans were purified using an Oasis HLB cartridge (60 mg/3 ml; Waters, Miford, Delaware, USA) and then were lyophilized. Permethylation was performed by using the solid NaOH technique.

### MALDI-TOF-MS analysis of permethylated *N*-glycans

Permethylated *N*-glycans were analyzed with a MALDI-TOF mass spectrometer in positive mode, controlled by the FlexControl 3.4 software (Bruker Daltonics, Karlsruhe, Germany). For MS analysis, the dried permethylated sample was resuspended in 10 *μ*l of acetonitrile. A total of 0.5 *μ*l of matrix solution (10 mg of 2, 5-DHB dissolved in 1 ml of 30% ethanol) and 0.5 *μ*l of the diluted analyte solution were spotted on the MALDI target plate (Bruker Daltonics). Then, the plate was analyzed by MS, whose spectra were obtained from Na^+^ adduct ions. MALDI-TOF-MS spectra were acquired using an UltrafleXtreme mass spectrometer (Bruker Corporation, Karlsruhe, Germany) in the positive-ion reflector mode, controlled by FlexControl 3.4 software Build 119 (Bruker Daltonics). Monosaccharide compositions were determined by blasting against database GlycoMod: http://www.expasy.ch/tools/glycomod/.

### Lentivirus production and infection

The ST8SIA4 shRNA sequences were inserted the pGLV3/H1/GFP+ Puro lentiviral plasmid. Lentivirus plasmids were prepared according to the manufacturer's instructions. For lentivirus infection, the cells were cultured in six-well tissue culture plates and infected the lentiviral vectors at a multiplicity of infection of 40 for 24 h. The medium was replaced with fresh complete medium. After 2 days, cells were observed by fluorescence microscopy to confirm that >90% of the cells were GFP positive. Subsequently, the GFP-positive cells were screened by the addition of 5 mg/l puromycin.

miR-26a/miR-26b/normal control (NC) mimics and miR-26a/miR-26b/NC inhibitors were chemically synthesized by Shanghai GenePharma Co. Ltd (Shanghai, China). MDA-MB231 cells were transfected with miRNAs (100 nM) using Lipofectamine 2000 Transfection Reagent (Invitrogen, Carlsbad, USA). Total RNA and protein were prepared after transfection and further used for quantitative real-time PCR or western blot analysis.

### Quantitative real-time PCR analysis

Total RNA was extracted from frozen tissues and breast cancer cell lines, using the RNeasy Mini Kit (Qiagen, valencia, CA, USA), and the purity of the preparation was checked by ratio of the absorbance at 260 and 280 nm. The cDNA was synthesized with 2 *μ*g of RNA using QuantiTect Reverse Transcription Kit (Qiagen). The expression of miR-26a/26b was determined by using mirVanaTM qRT-PCR microRNA Detection Kit (Ambion Inc., Austin, TX, USA) and normalized using U6 snRNA and RNU48 as control. ST mRNA was quantified with SYBR Green Quantitative Real-Time PCR Master Mix Kit (Toyobo Co., Osaka, Japan) and normalized to GAPDH and *β*-action, respectively. The expression level of ST target genes was determined by using Biosystems 7300 Real-Time PCR System (ABI, Foster City, CA, USA). The sequences of primers were as shown in Table 2. Three independent experiments were each performed in triplicate.

### Protein extraction and western blot

Cells were washed two times cold phosphate-buffered saline (PBS). Total protein was extracted using cell lysis buffer and a protein concentration determined with the BCA Protein Assay Kit (Pierce). Twenty micrograms for each sample were separated in 10% SDS-PAGE and then transferred a polyvinylidene difluoride membrane (Pall Corporation, New York, USA). The membrane was blocked with 5% non-fat dry milk in PBS containing 0.1% Tween 20 (PBST) for 1 h and then probed with anti-ST8SIA4 monoclonal antibody (Abcam, Cambridge, UK; 1:1000 dilution) Detection was achieved using a secondary anti-rabbit HRP-conjugated IgG (Santa Cruz Biotechnology; 1 : 1000 dilution). GAPDH was used as a control. Immunoreactive proteins were visualized using ECL Western Blotting Kit (Amersham Biosciences, Beijing, China). Membranes were incubated with anti GAPDH diluted 1 : 1000 (Santa Cruz Biotechnology) as an endogenous control.

### CCK-8 assays

Cell proliferation was measured using a CCK-8 (KeyGen, Nanjing, China) according to the manufacturer's instruction. Cells (1 × 10^3^/well) were seeded into 96-well plates with 100 *μ*l of DMEM medium containing 10% FBS and cultured in a humidified incubator (at 37 °C with 5% CO_2_) for 24, 48 and 72 h. Then, each well was added with 10 *μ*l CCK-8 solutions at 37 °C for 2 h. The absorbance at 450 nm was immediately measured using a microplate reader (iMark; Bio-Rad Laboratories Inc., Hercules, CA, USA). Each experiment was performed at least three times.

### Wound-healing assay

Wound-healing assay was carried out to determine the cell protrusion and migration ability of tumor cells. Briefly, 1 × 10^4^ cells were seeded into 12-well dishes and grew until 70–80% confluence. Sterilized a P200 pipette tip was used to inflict wounding across the cell monolayer, and the debris was washed with PBS. Migration of cells into the wound was then observed at different time. Cells migrated into the wounded area or protruded from the border of the wound were visualized and photographed under the inverted microscope at different times. At least eight fields for each condition were taken randomly in each well by a × 100 magnification and cells in three wells of each group were quantified in each experiment. Experiments were carried out in triplicate at least three times.

### Cell invasion assay

Cell invasion were measured using 8-*μ*m pore uncoated or ECMatrix gel (Chemicon)-coated transwell inserts, respectively (Trevigen, City of Gaithersburg, MD, USA). Briefly, cells (1 × 10^5^) were harvested in serum-free DMEM and added to the upper chamber. Medium containing 10% FCS was added to the bottom chamber, and cells were allowed to invade for 24 h at 37 °C, and cells were allowed to invade for 24 h at 37 °C. At the end of incubation, the invaded cells at the reverse side of the insert were fixed in methanol and were stained with Wright–Giemsa. The number of invaded cells on the lower side of the membrane was counted at × 400 magnification from 10 different fields of each filter. Each experiment was performed in triplicate and repeated three times.

### Immunofluorescence analysis

Cells were transfected with miRNA mimic or miRNA inhibitor as described above and cultured in glass coverslips. Cells were then washed with PBS and subsequently fixed with cold 4% formaldehyde for 10 min at room temperature. Following fixation, cells were permeabilized with PBS containing 0.3% Triton X-100 and 2% BSA for 30 min at room temperature. Primary antibody Ki67 (Abcam, Cambridge, MA, USA) or ST8SIA4 was diluted 1 : 200 in 5% BSA in PBS and incubated on slides overnight at 4 °C. Slides were then washed three times with PBS and incubated 1 h with secondary antibody. After two washes with PBS, slides were incubated for 10 min with 4, 6-diamino-2-phenylindole (DAPI; Sigma-Aldrich, St. Louis, MO, USA) in PBS for nuclear staining. Images were taken in a Carl Zeiss fluorescence microscopy (Carl Zeiss, Hallbergmoos, Germany).

### Tumorigenicity assays in nude mice

Female athymic nude mice, aged 4–6 weeks, were obtained from the Animal Facility of Dalian Medical University, and were used for subcutaneous tumor implantation. Approximately 1 × 10^7^ cells were injected subcutaneously into the right flank of each nude mouse, respectively. Measurements of nodules began in the first week of postinoculation and monitored weekly by measuring the perpendicular tumor diameters, length (*L*) and width (*W*), with a vernier caliper. All mice were killed according to the established humane end points under anesthesia 4 weeka after injection. Mouse tumors were isolated, photographed and weighed. Tissues were fixed in 10% buffered formalin, processed and embedded in paraffin. Tumor size was measured using calipers, and tumor volume (mm^3^) was estimated by 1/2(length × width^2^).

### Immunohistochemistry

The breast cancer tissue of patients and xenograft tumor of mouse were fixed in 4% paraformaldehyde, dehydrated in a graded series of alcohol and then embedded in paraffin. Sections (4 *μ*m thick) were deparaffinized, rehydrated and then immersed in 3% hydrogen peroxide for 10 min to block endogenous peroxidase. After consecutive washing with PBS, the slices were incubated with primary anti-ST8SIA4 or Ki67 antibody (1:200; Abcam, Cambridge, UK) at 4 °C overnight. The secondary streptavidin-HRP-conjugated antibody staining (1 : 1000; Santa Cruz Biotechnology) was performed at room temperature for 60 min. Slides were then washed in PBS and developed applying DAB for 5 min. Finally, the sections were counterstained with hematoxylin and coverslipped. Negative controls, without antibodies were included. All stained sections were examined with light microscopy and reviewed by three observers.

### Dual-luciferase assays

For luciferase reporter experiments, a pmirGLO Dual-Luciferase miRNA Target Expression Vector was chosen (Promega, Madison, WI, USA). Plasmids containing WT ST8SIA4 and mutant ST8SIA4 3′-UTR were specifically synthesized (Promega). Firefly luciferase and *Renilla* luciferase functioned as a tracking gene in these plasmids. For the reporter assays, 5 × 10^4^ HEK 293 T cells were seeded in 24-well plates at 80% confluence and cultured for 24 h. The mimics of miRNA or miR-NC along with firefly luciferase reporter gene construct and a Renilla luciferase construct were co-transfected per well. After 48 h, the activities of firefly and *Renilla* luciferases were measured using the Dual-Luciferase Assay Kit (Promega) and normalized to those of *Renilla* luciferase activities. The mean of relative luciferase activities from the cells transfected with the miR-control was set at 100. All experiments were performed in triplicate. The results were statistically analyzed using Student's *t*-test.

### Statistical analysis

Data are mean±S.D. of at least three independent experiments. Student's *t*-test was applied to determine differences between samples. The paired *t*-test was used for comparison between cancer and adjacent tissues. All analyses were performed using SPSS 17.0 statistical packages (SPSS Inc., Chicago, IL, USA). A value of *P<0.05 was considered to be statistically significant.

## Figures and Tables

**Table 1:** Composition of permethylated *N*-glycans released from breast cancer cell lines and breast cancer patients determined by MALDI-TOF MS analysis

**Glycan number**	**Observed *m*/*z***				**Chemical composition**
	**MDA-MB-231**	**MCF-10A**	**Tumor**	**Adjacent**	
1	1345.67	1345.65	1345.64	1345.66	(Fuc)+(Man)_3_(HexNAc)_2_
2	No	No	1375.64	1375.67	(Hex)+(Man)_3_(HexNAc)_2_
3	No	No	1416.67	No	(HexNAc)+(Man)_3_(HexNAc)_2_
4	1579.77	1579.74	1579.73	1579.77	(Man)_2_+(Man)_3_(HexNAc)_2_
5	1590.78	1590.76	1590.74	No	(HexNAc)(Fuc)+(Man)_3_(HexNAc)_2_
6	No	No	1620.76	No	(Hex)(HexNAc)+(Man)_3_(HexNAc)_2_
7	No	No	1753.81	No	(Hex)_2_(Fuc)+(Man)_3_(HexNAc)_2_
8	1783.86	1783.84	1783.82	1783.86	(Man)_3_+(Man)_3_(HexNAc)_2_
9	No	No	1794.84	1794.88	(Hex)(HexNAc)(Fuc)+(Man)_3_(HexNAc)_2_
10	1824.88	1824.86	1824.84	1825.16	(Hex)_2_(HexNAc)+(Man)_3_(HexNAc)_2_
11	1835.91	1835.87	1835.86	No	(HexNAc)_2_(Fuc)+(Man)_3_(HexNAc)_2_
12	No	No	1865.86	No	(Hex)(HexNAc)_2_+(Man)_3_(HexNAc)_2_
13	1987.96	1987.93	1987.92	1987.95	(Man)_4_+(Man)_3_(HexNAc)_2_
14	No	No	2028.94	No	(Hex)_3_(HexNAc)+(Man)_3_(HexNAc)_2_
15	2070.01	2069.99	2069.97	No	(Hex)_2_(HexNAc)_2_+(Man)_3_(HexNAc)_2_
16	2081.03	2081.01	2080.97	No	(HexNAc)_3_(Fuc)+(Man)_3_(HexNAc)_2_
17	2192.06	2192.04	2192.02	2192.05	(Man)_5_+(Man)_3_(HexNAc)_2_
18	2244.11	2244.07	2244.05	2244.10	(Hex)_2_(HexNAc)_2_(Fuc)+(Man)_3_(HexNAc)_2_
19	2285.13	2285.11	2285.05	2285.12	(Hex)(HexNAc)_3_(Fuc)+(Man)_3_(HexNAc)_2_
20	2396.16	2396.13	2396.11	2396.15	(Man)_6_+(Man)_3_(HexNAc)_2_
21	2418.18	2418.14	2418.12	2418.18	(Hex)_2_(HexNAc)_2_(Fuc)_2_+(Man)_3_(HexNAc)_2_
22	2431.19	No	No	No	(Hex)_2_ (HexNAc)_2_(NeuAc)+(Man)_3_(GlcNAc)_2_
23	2472.16	2472.13	2472.10	2472.22	(Hex)(HexNAc)_3_(NeuAc)+(Man)_3_(GlcNAc)_2_
24	2489.24	2489.19	2489.16	2489.22	(Hex)_2_(HexNAc)_3_(Fuc)+(Man)_3_(HexNAc)_2_
25	2605.27	2605.24	2605.21	2605.27	(Hex)_2_(HexNAc)_2_(Fuc)(NeuAc)+(Man)_3_(HexNAc)_2_
26	2693.33	2693.30	2693.25	2693.31	(Hex)_3_(HexNAc)_3_(Fuc)+(Man)_3_(HexNAc)_2_
27	2779.36	2779.33	2779.29	2779.35	(Hex)_2_(HexNAc)_2_(Fuc)_2_(NeuAc)+(Man)_3_(HexNAc)_2_
28	2867.42	2867.37	2867.34	No	(Hex)_3_(HexNAc)_3_(Fuc)_2_+(Man)_3_(HexNAc)_2_
29	2880.40	No	No	No	(Hex)_3_(HexNAc)_3_(NeuAc)+(Man)_3_(HexNAc)_2_
30	No	2938.43	2938.39	2938.45	(Hex)_3_(HexNAc)_4_(Fuc)+(Man)_3_(HexNAc)_2_
31	2966.45	2966.40	No	No	(Hex)_2_(HexNAc)_2_(Fuc)(NeuAc)_2_+(Man)_3_(HexNAc)_2_
32	3054.49	3054.47	3054.45	3054.47	(Hex)_3_(HexNAc)_3_(Fuc)(NeuAc)+(Man)_3_(HexNAc)_2_
33	3142.55	3142.52	3142.51	3142.55	(Hex)_4_(HexNAc)_4_(Fuc)+(Man)_3_(HexNAc)_2_
34	3415.67	3415.66	No	No	(Hex)_3_(HexNAc)_3_(Fuc)(NeuAc)_2_+(Man)_3_(HexNAc)_2_
35	3503.76	3503.71	3503.70	No	(Hex)_4_(HexNAc)_4_(Fuc)(NeuAc)+(Man)_3_(HexNAc)_2_
36	3591.81	3591.78	3591.77	No	(Hex)_5_(HexNAc)_5_(Fuc)+(Man)_3_(HexNAc)_2_
37	3776.93	No	No	No	(Hex)_3_(HexNAc)_3_(Fuc)(NeuAc)_3_+(Man)_3_(GlcNAc)_2_
38	3864.98	3864.91	No	No	(Hex)_4_(HexNAc)_4_(Fuc)(NeuAc)_2_+(Man)_3_(GlcNAc)_2_
39	3953.07	3953.04	No	No	(Hex)_5_(HexNAc)_5_(Fuc)(NeuAc)+(Man)_3_(HexNAc)_2_
40	4226.21	No	No	No	(Hex)_4_(HexNAc)_4_(Fuc)(NeuAc)_3_+(Man)_3_(GlcNAc)_2_

Abbreviation: MALDI-TOF MS, matrix-assisted laser desorption/ionization-time of flight mass spectrometry

**Table 2:** qRT-PCR conditions and primer sequences for analysis of gene expression

**Gene**	**Primers**	**Amplicon (bp)**
*ST3GAL1*	5'-CAGAGATGGACGGTCACT-3′ 5′-CAACTGTGGTTTCTGACG-3′	197
*ST3GAL2*	5′-GTGCCTCCGACTGGTTTG-3′ 5′-GAAGCGGTAGGGGTTCTC-3′	191
*ST3GAL3*	5′-TATGCTTCAGCCTTGATG-3′ 5′-TTGGTGACTGACAAGATGG-3′	164
*ST3GAL4*	5′-ATGTTGGCTCTGGTCCTG-3′ 5′-AGGAAGATGGGCTGATCC-3′	176
*ST3GAL5*	5′-CAAAGCAAGATGAGAAGG-3′ 5′-AAACTTGGGACGACATTC-3′	213
*ST3GAL6*	5′-TATTATGGGGAACGAATG-3′ 5′-AAAAGGGTGAAACTGATG-3′	195
*ST6GAL1*	5′-CTTGTTTTCCTGCTCAGA-3′ 5′-GCAAACAGAAGAAAGACCA-3′	166
*ST6GAL2*	5′-ACGCTGCTGATTGACTCTTCT-3′ 5′-CACATACTGGCACTCATCTAA-3′	160
*ST6GALNAC1*	5′-CTGGTCTTCTTTCTCTTCG-3′ 5′-GTTGAGGGCATTGTTCTCT-3′	192
*ST6GALNAC2*	5′-CTTTGCCCTGTACTTCTCG-3′ 5′-CAGCACTGGAATGGAGAGA-3′	205
*ST6GALNAC3*	5′-GGACAACCTGGTACAAAGT-3′ 5′-TATCTCATTTCCCACCTTC-3′	174
*ST6GALNAC4*	5′-ACCTGCCTGGACCACCACT-3′ 5′-TCGGCACTGTCGATCTCAG-3′	188
*ST6GALNAC5*	5′-TGGACGGATACCTCGGAGT-3′ 5′-GTCTGGTCAATCTGGGAGC-3′	121
*ST6GALNAC6*	5′-ACCTACCCCTCAGCAGACG-3′ 5′-CTTGAGGTTGACAGGTCGG-3′	179
*ST8SIA1*	5′-TACTCTCTCTTCCCACAGG-3′ 5′-GACAAAGGAGGGAGATTGC-3′	149
*ST8SIA2*	5′-GTGGTCTTCCTCATCTTCG-3′ 5′-GAGGAGCCGTTTATTACAAC-3′	140
*ST8SIA3*	5′-ATTCTCTCACCCAGGAACTC-3′ 5′-CAATCCGAACACTATTCTTG-3′	141
*ST8SIA4*	5′-CAAGAACTGAGGAGCACC-3′ 5′-TTTCCAACCTTCTACATTGTG-3′	140
*ST8SIA5*	5′-CCTTTGCCTTGGTGACCT-3′ 5′-CATGGACAGCACCTTCACT-3′	152
*ST8SIA6*	5′-CGGCAAGCAGAAGAATATG-3′ 5′-GCTTTCCACCTCGTAACTC-3′	126
*GAPDH*	5′-CTCCTCCACCTTTGACGCTG-3′ 5′-TCCTCTTGTGCTCTTGCTGG-3′	175

Abbreviation: qRT-PCR, quantitative reverse transcription-PCR
