# Supplementary material for: Functional roles of sialylation in breast cancer progression through miR-26a/26b targeting ST8SIA4
Source: Cell Death Dis. 2016 Dec 29;7(12):e2561–. doi: 10.1038/cddis.2016.427 (PMC5260976; doi:10.1038/cddis.2016.427)
Supplement: Supplementary Table 1 [file cddis2016427x1.doc]

**Supplemental Table 1.** Clinicopathological characteristics of breast cancer patients

| **Characteristics** | n |
| --- | --- |
| **Group** |  |
| Cancer tissues | 29 |
| Transitional tissues | 29 |
| **Age(years)** |  |
| ≥48 | 16 |
| <48 | 13 |
| **TNM stage** |  |
| I | 17 |
| II | 12 |
| **Histological grade** |  |
| G1 | 15 |
| G2 | 14 |
| **Lymph node status** |  |
| Negative | 18 |
| Positive | 11 |
| **ER expression** |  |
| Negative | 7 |
| Positive | 22 |
| **PR expression** |  |
| Negative | 9 |
| Positive | 20 |
| **HER2 expression** |  |
| Negative | 24 |
| Positive | 5 |
