# Supplementary figures and images for: Functional roles of sialylation in breast cancer progression through miR-26a/26b targeting ST8SIA4
Source: Cell Death Dis. 2016 Dec 29;7(12):e2561–. doi: 10.1038/cddis.2016.427 (PMC5260976; doi:10.1038/cddis.2016.427)

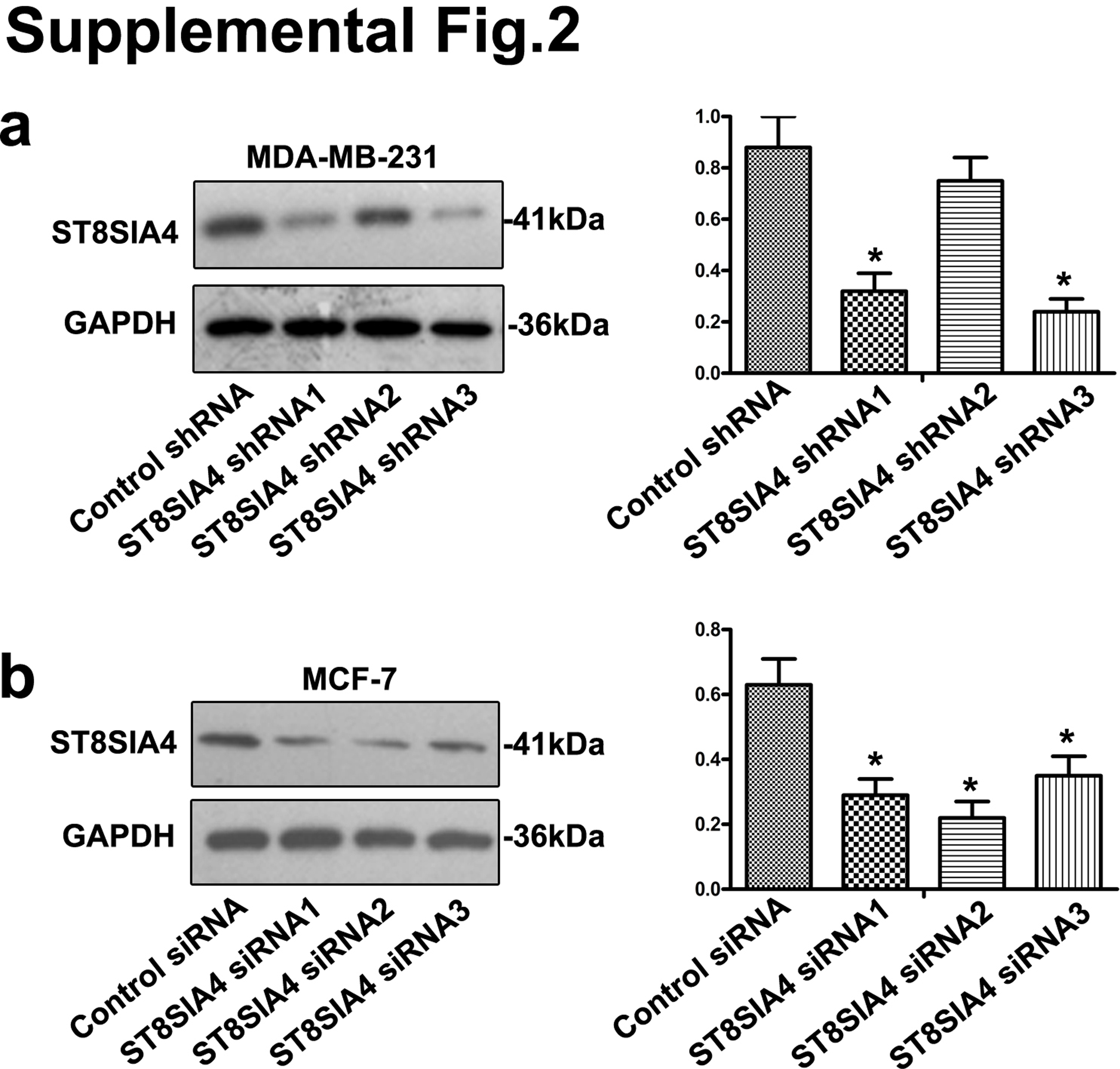

Supplement: Supplementary Figure 2 [file cddis2016427x3.tif]
